# Prevalence of antimicrobial resistant pathogens from blood cultures: results from a laboratory based nationwide surveillance in Ghana

**DOI:** 10.1186/s13756-017-0221-0

**Published:** 2017-06-13

**Authors:** Japheth Awuletey Opintan, Mercy Jemima Newman

**Affiliations:** Medical Microbiology Department, School of Biomedical and Allied Health Sciences, Korle-Bu, P. O. Box KB 4236, Accra, Ghana

**Keywords:** Bloodstream infections, Antimicrobial drug resistance, Blood culture, Surveillance, Methicillin resistance

## Abstract

**Background:**

Blood stream infections (BSI) are critical medical conditions with high morbidity and mortality. There is paucity of information on BSI from surveillance studies in Ghana.

**Aim:**

This study sought to demonstrate how useful BSI data can be gleaned from population-based surveillance, especially from resource-limited settings.

**Methods:**

Data from a nationwide surveillance of antimicrobial drug resistance (AMR) in Ghana were extracted and analyzed. Secondly, we revived archived *Staphylococcus aureus* isolates from blood cultures that were cefoxitin resistant (CRSA), and screened these for protein A (*spa*) and *mec* A genes.

**Results:**

Overall blood culture positivity was 11.2% (714/6351). All together, participating laboratories submitted 100 multidrug resistant blood culture isolates (Gram-negative = 49 and Gram-positive = 51). Prevalence of some Gram-negative isolates was as follows; *Escherichia coli* (20.4%), *Pseudomonas aeruginosa* (16.3%), *Enterobacter* spp. (14.3%), *Salmonella* serotype Typhi (8.2%) and Non-typhoidal *Salmonella* [NTS] (8.2%). Gram-positive pathogens included *Staphylococcus aureus* (66.7%), coagulase negative *S. aureus* [CoNS] (17.6%) and *Streptococcus pneumoniae* (11.8%). No methicillin resistance was confirmed in our CRSA isolates. Most blood stream associated infections were from inpatients (75%) and cultured bacteria were resistant to common and cheaper antimicrobials.

**Conclusion:**

*E. coli* and *S. aureus* are common pathogens associated with BSI in Ghana and they are resistant to several antimicrobials. Active and continuous AMR surveillance can serve multiple purposes, including data generation for BSI.

## Background

Bloodstream infections (BSI) are important cause of morbidity and mortality, that require prompt and appropriate empiric therapy before blood culture results are ready. The management of BSI is further complicated in an era of increasing antimicrobial resistance (AMR) [1]. Empiric antimicrobial therapy when administered early in the course of BSI has the potential to save lives [2]. However, the epidemiology of the causative agents are not static, constantly changing over time [3]. Especially in resource limiting settings with inadequate laboratory support for culture and antimicrobial surveillance, BSI management is further complicated.

Several resource-rich countries have surveillance systems in place to monitor changing trends of infections including BSI. Examples include the Health Protection Agency (HPA) in England [3] and the Canadian Antimicrobial Resistance Alliance (CARA) [4]. These surveillance systems readily provide useful data on microorganisms of clinical significance, including those associated with BSI, providing clues on aetiological trends and AMR. Active surveillance systems are non-existent in most resource-limited countries including Ghana, in spite of the high disease in these countries. Several local studies, especially in tertiary care facilities have been done in Ghana and elsewhere, but these are either disease condition or age specific [5–8]. In a previous study, we presented the overall results of a nationwide surveillance. In that study, only about 40% of participating hospitals provided data on blood culture results. In the present study, we focused on blood stream infections and analyzed the data on blood culture results that we did receive by those 40% of participating hospitals. We did this to emphasize the importance of more detailed information that can be obtained from more detailed reporting, and to encourage better participation in such nationwide surveillance.

## Methods

Data on bloodstream infections (BSI), including patient information, bacterial isolates and their antibiogram were electronically extracted from our surveillance records [9]. The Department of Medical Microbiology, School of Biomedical and Allied Health Sciences (DM-SBAHS) coordinated this surveillance, June–November 2014. Participating laboratories used in-house standard operating procedures to culture and to identify bacteria from all specimens submitted for bacteriological analysis. Antimicrobial susceptibility testing was done by the disc diffusion test, in accordance with the Clinical and Laboratory Standards Institute [10] guidelines. Multiple drug resistant isolates, defined as resistance to more than two antimicrobial drug classes were submitted to DM-SBAHS. The location and description of participating laboratories are described elsewhere [9] and included Teaching, Regional, District and Faith-based hospitals in Ghana.

We analyzed data on all blood cultures submitted by participating laboratories to DM-SBAHS. To confirm methicillin resistant *Staphylococcus aureus* (MRSA), all stored cefoxitin-resistant *S. aureus* (CRSA) isolates were revived on blood agar plates. The identities of these CRSA were reconfirmed by Gram stain, catalase and tube coagulase tests. Genomic DNAs were extracted from pure CRSA isolates and were then screened by polymerase chain reaction for *Staphylococcus*-specific genes (*spa* and *mecA*), using previous protocols [11]. Data on BSI were stratified by age, isolate source and hospital facility and were summarized in tables or percentages.

## Results

In total, 11 of 18 (61.1%) laboratories processed and submitted surveillance data on blood cultures. The overall culture positivity rate was 11.2% (714/6351) with great variabilities ranging between 4.7–27.3% (Table 1). Total number of non-duplicate multidrug resistant blood isolates received from participating laboratories was 100 (Gram-negative, *n* = 49; Gram-positive, *n* = 51). Blood culture Gram-negative (GN) isolates included *Escherichia coli* (20.4%), *Pseudomonas aeruginosa* (16.3%), *Enterobacter* spp. (14.3%), *Salmonella* Typhi (8.2%) and Non-Typhoidal Salmonella [NTS] (8.2%). *E. coli* was predominantly isolated from patients aged older than 10 years. Other GN rods like typhoidal and NTS were mostly recovered from children less than 10 years of age [Table 2]. Gram-positive (GP) isolates included *Staphylococcus aureus* (66.7%), coagulase negative *Staphylococcus* [CNS] (17.6%) and *Streptococcus pneumoniae* (11.8%). *Staphylococus* and *Streptococcus* were generally recovered from children less than 10 years. However many patients with staphylococcal associated BSI had missing data for age (Table 2). Multidrug resistant isolates were predominately recovered from inpatients (>70%). Tables 3 and 4 show the prevalence of antimicrobial drug resistance of epidemiologically important GN and GP bacterial isolates, respectively.

**Table 1:** Blood cultures processed and data submitted during surveillance

Laboratory code	Total blood cultures processed	Positives, rates (%)	Multi-drug resistant isolates submitted during surveillance
BaS	315	86 (27.3)	41
AcL	226	54 (23.9)	16
WsE	505	43 (8.5)	14
NoT	1376	102 (7.4)	13
AsK	3610	398 (11.0)	7
CeU	13	3 (23.1)	1
AsO	146	16 (10.9)	1
BaB	21	1 (4.7)	1
EaK	95	8 (8.4)	1
UeB	44	3 (6.8)	1
Total	6351	714	100

Teaching hospitals: Tamale (NoT) and Komfo Anokye (AsK). Regional hospitals: Sunyani (Bas), Eastern (EaK) and Upper East (UeB). District hospital: LEKMA (AcL), Faith base: St Patrick, Offinso (AsO), St Patrick Holy Family, Berekum (BaB). Quasi-government hospital: University, Cape Coast (CeU). Zonal public health reference laboratory: Sekondi (WsE)

**Table 2:** Gram negative and positive multiple drug resistant isolates from surveillance records

Bloodstream isolates	n (%)	Isolate source			Age categories/years					
		In-patient	Out-patient	Not indicated^a^	< 1	1–10	11–30	31–50	> 50	Not indicated^a^
Gram negatives (*n* = 49)										
*Escherichia coli*	10	9	1	-	1	-	3	2	2	2
*Salmonella* Typhi	4	2	2	-	-	4	-	-	-	-
Non-Typhoidal Salmonella	4	4	-	-	2	2	-	-	-	-
*Enterobacter* spp	7	6	1	-	3	-	1	1	-	2
*Serratia marcescens*	4	4	-	-	-	2	-	-	1	1
*Serratia* spp	2	2	-	-	-	2	-	-	1	1
*Klebsiella pneumoniae*	2	2	-	-	-	2	-	-	-	-
*Citrobacter korseri*	1	-	1	-	1	-	-	-	-	-
*Citrobacter* spp	5	5	-	-	2	3	-	-	-	-
*Acinetobacter*	1	1	-	-	1	-	-	-	-	-
*Pseudomonas aeruginosa*	8	6	1	1	3	1	-	1	2	1
*Proteus mirabilis*	1	1	-	-	-	-	1	-	-	-
Gram positives (*n* = 51)										
*Staphylococcus aureus*	34	23	8	3	11	10	3	1	2	7
CNS	9	8	-	1	3	2	1	-	1	2
*Streptococcus pneumoniae*	6	5	1	-	2	3	-	-	-	1
*Streptococcus pyogenes*	1	1	-	-	1	-	-	-	-	-
*Enterococcus* spp	1	-	-	-	-	-	1	-	-	-
Totals	100	75	15	5	30	29	10	5	8	16

CNS*, Coagulase negative Staphylococcu*

-indicates not applicable

^a^unavailable date

**Table 3:** Antibiogram of epidemiologically important Gram negative blood culture isolates

Antimicrobial agent	*E. coli* (*n* = 10)	*S. Typhi* (*n* = 4)	NTS (*n* = 4)	*Pseudomonas* (*n* = 8)	*Enterobacter (n = 7)*
	n, (% resistant)	n, (% resistant)	n, (% resistant)	n, (% resistant)	n, (% resistant)
Ampicillin	10 (100)	4 (100)	3 (100)	8 (62.5)	7 (100)
Cefuroxime	8 (87.5)	3 (66.6)	3 (50)	6 (66.7)	7 (85.7)
Cefotaxime	9 (88.9)	4 (50)	3 (60)	8 (87.5)	7 (85.7)
Meropenem	8 (12.5)	3 (0)	4 (0)	5 (20)	5 (0)
Amikacin	8 (0)	2 (0)	2 (0)	8 (12.5)	3 (33.3)
Gentamicin	10 (30)	4 (25)	3 (50)	8 (25)	5 (80)
Ciprofloxacin	10 (60)	2 (100)	ND	8 (12.5)	4 (25)
Levofloxacin	7 (42.9)	2 (50)	ND	ND	ND
Trimethoprim/Sulfamethoxazole	10 (100)	4 (50)	3 (100)	8 (75)	7 (71.4)
Chloramphenicol	9 (100)	4 (50)	3 (100)	7 (71.4)	7 (57.2)
Tetracycline	10 (100)	4 (50)	3 (50)	8 (62.5)	5 (80)

*NTS* Non-typhoidal Salmonella, *ND* indicates not done

**Table 4:** Antibiogram of epidemiologically Gram positive blood culture isolates

Antimicrobial agent	*Staph aureus* (*n* = 34)	CNS (*n* = 9)	*S. pneumoniae* (*n* = 6)
	n, % resistance	n, % resistance	n, % resistance
Penicillin	26 (23.1)	6 (0)	6 (0)
Ampicillin	30 (100)	8 (100)	6 (0)
Cloxacillin	14 (100)	4 (0)	ND
Flucloxacillin	17 (14.3)	1 (0)	ND
Augmentin	12 (75)	ND	ND
Cefuroxime	26 (53.8)	6 (16.7)	ND
Cefotaxime	14 (71.4)	8 (62.5)	ND
Gentamicin	30 (30)	8 (25)	6 (16.7)
Ciprofloxacin	29 (34.5)	8 (62.5)	6 (50)
Trimethoprim/Sulfamethoxazole	30 (70)	8 (100)	6 (0)
Erythromycin	5 (64)	8 (83.3	6 (66.7)
Tetracycline	31 (77.5)	8 (100)	6 (83.3)
Vancomycin	15 (0)	ND	2 (0)

*CNS* Coagulase negative Staphylococcus, *ND* indicates not done

In total, 4 of 6 CRSA were successfully revived and molecularly screened for MRSA (*mecA* and *spa* genes). None of these tested positive for MRSA, both negative for *mecA* and *spa* genes.

## Discussion

To inform empiric antibiotic guidelines at institutional level, one requires regular review of pathogen spectrum and antibiogram. Critical medical information to manage BSI could be gleaned from population-based or nosocomial surveillance systems [6, 8, 12–16]. However, the current trend for investigating BSI is to use population-based approaches [15, 17]. Information from such systems becomes even more useful when they are designed to capture additional indicators. For example, illness severity as indicated by Pitt bacteraemia score, [18] receipt of adequate antimicrobial regimen, [19] primary source of infection, and the presence or absence of malignancies [20]. Previous studies on BSI in Ghana were limited to tertiary facilities and restricted to particular age category [8, 16, 21, 22]. The current study used population-based surveillance to investigate BSI.

Overall blood culture positivity rate was only about 11% with a wide variability range (between 4.7% to 27%) across the 10 surveillance sites in Ghana. These rates have serious implications on patient care and also reflect blood culture capabilities in the respective hospitals. A combination of the following reasons may apply; some patients already on antibiotics prior to specimen collection, misdiagnosis from clinicians or laboratories using inadequate culture methods. It is worth noting that most of the regional hospitals in this study use the BACTEC blood cultures systems, but are usually short of one supply or the other. Alizadeh and colleagues however showed that both BACTEC and conventional blood culture methods have high validity [23]. We propose that the BACTEC and conventional blood culture methods should be used complementarily, especially in resource-limited countries, where consumables for the BACTEC are often in short supply. Infrastructure for blood culture services, whether BACTEC or conventional should be available for BSI diagnosis and management. Several studies in Ghana [8, 22] had blood culture positivity rates greater than 20% and in a large Indian study with over 135,000 cultures, a 14% rate was observed [24]. In a meta-analysis review, Reddy and colleagues identified bloodstream infections in 13.5% and 8.2% adults and children, respectively, with overall in-hospital case fatality rate of about 18% [25]. Our analysis did not follow up on patient outcomes but poor laboratory infrastructure and inadequate diagnostics, often results in poor treatment outcomes, sometimes confusing malaria bloodstream infections with other pathogens [25].

We found slightly more Gram-positive bacteria (GPB) in association with BSI in the current study, contrary to the Gram-negative bacteria (GNB) observed in several studies in Ghana [8, 22] and elsewhere [26]. Among the GNB, *E coli* was responsible for about a fifth of the BSI in the current study. Elsewhere, patient specific studies have generally associated *E. coli* to BSI [6, 8, 22]. On the contrary, in Europe, though *E coli* was previously topping the list of organisms associated with BSI, this has currently given way to MRSA [3, 14]. *E coli* associated BSI in most cases is secondary to focal infections such as urinary tract, abdominal, hepato-biliary sepsis, or surgical site infections [3]. An association with central vascular lines has also been reported [13]. Besides *E. coli*, *S. Typhi* and NTS were other GNB with higher prevalence, especially among children with ages less than 10 years. Compared to resource-rich countries where *Salmonella* (whether typhoidal or nontyphoidal) is almost never implicated in BSI [3, 4, 14], persistently, this organism is a common cause of GN sepsis in resource limiting countries [7, 8, 22, 24, 27]. It is generally difficult to distinguish between community and hospital acquired BSI. However, *Pseudomonas* is often associated with hospital-acquired BSI, often related to indwelling devices. In the current study *Pseudomonas* was responsible for more than 16% of all GN BSI.

Among the GPB, *Staphylococcus* and CNS were the predominant organisms, together accounting for greater than 80% of all GP sepsis. *Staphylococcus* was found more in patients less than 30 years of age, whilst CNS was found in children less than 10 years. Higher frequencies of GPB have been implicated in some earlier BSI studies from the USA and Canada [14]. GPB dominates in places where more prosthetic devices, mainly intravenous catheters, invasive procedures and specific procedures are done [28]. We did not confirmed MRSA in the current study. Meanwhile, studies from the largest tertiary hospital in Ghana have reported MRSA, primarily based on cefoxitin resistance [8, 22]. This tertiary hospital did not submit data for the current surveillance. MRSA associated BSI has been reported elsewhere. In England, mandatory and voluntary surveillance systems helped in reducing high MRSA bacteraemia [29]. *Streptococcus pneumoniae*, the next frequent GPB were predominantly found in children less than 5 years of age, comparable to other observations [8].

The high prevalence of antimicrobial drug resistance (AMR) observed in the GNB associated with BSI is alarming. Among the GNB, it appears meropenem and amikacin are the last resort antimicrobials. The hospitals in our surveillance reported and submitted data only on multi-drug resistant isolates, and we did not have information on the isolates susceptible. However, management of BSI in these patients with these high resistant bacteria will be difficult and may have bad outcomes. Extended spectrum beta-lactamase (ESBLs) and carbapenemase producing enterobacteriaceae (CRE) isolates in the current analysis is a worrying concern. These hydrolyzing enzymes render these antimicrobials ineffective, but are sparingly screened for in Ghana, especially, in bloodstream infections [8, 30]. Elsewhere, relatively high levels of these enzymes have been found in bloodstream isolates [31]. Scaling up of laboratory capacity in resource-limited countries like Ghana is urgently needed. In the absence of molecular assays for these enzymes, phenotypic assays would be helpful for patient management. GPB like *Staphylococcus* and CNS were also resistant to older antibiotics. We did not observe any MRSA (by molecular assay) and vancomycin resistant *Staphylococcus* in the current study, contrarily to other investigations in Ghana [8]. However, it is important to highlight that all *Staphylococcus aureus* isolates submitted by participating laboratories were resistant to cloxacillin (Table 4). Since cefoxitin is a better inducer for *mecA* (MRSA), it is currently preferred to oxacillin. A polymerase chain reaction (PCR), targeting the *mecA* gene remains the gold standard for MRSA confirmation [32]. However, PCR is seldom used in routine clinical laboratories because of logistics and inability to standardized laboratory protocols. The current study is not without limitations, which includes incomplete data sets and detailed clinical information. No clinical information was available about the patients from the participating laboratories. It is therefore difficult to differentiate between community acquired and hospital acquired BSIs [15, 17]. Complete clinical information, if available will help in ruling out some possible skin contaminants, which many include some CNS. Information on blood culture contaminants has implication on cost and practice. Finally, the relatively fewer numbers of bacterial isolates tested in any particular species limits the power of our study.

## Conclusion

*E. coli* and *Salmonella* (typhoidal and NTS), *Staphylococcus aureus*, CNS and *Streptococcus pneumoniae* are often associated with BSI in Ghana. Most of these organisms are resistant to common antibiotics. Active and continuous surveillance systems will play a dual role in monitoring BSI and AMR.
